# Alzheimer's Disease and the Amyloid Cascade Hypothesis: A Critical Review

**DOI:** 10.1155/2012/369808

**Published:** 2012-03-17

**Authors:** Christiane Reitz

**Affiliations:** ^1^Taub Institute for Research on Alzheimer's Disease and the Aging Brain, College of Physicians and Surgeons, Columbia University, New York, NY 10032, USA; ^2^Gertrude H. Sergievsky Center, College of Physicians and Surgeons, Columbia University, New York, NY 10032, USA; ^3^Department of Neurology, College of Physicians and Surgeons, Columbia University, New York, NY 10032, USA

## Abstract

Since 1992, the amyloid cascade hypothesis has played the prominent role in explaining the etiology and pathogenesis of Alzheimer's disease (AD). It proposes that the deposition of *β*-amyloid (A*β*) is the initial pathological event in AD leading to the formation of senile plaques (SPs) and then to neurofibrillary tangles (NFTs), neuronal cell death, and ultimately dementia. While there is substantial evidence supporting the hypothesis, there are also limitations: (1) SP and NFT may develop independently, and (2) SPs and NFTs may be the products rather than the causes of neurodegeneration in AD. In addition, randomized clinical trials that tested drugs or antibodies targeting components of the amyloid pathway have been inconclusive. This paper provides a critical overview of the evidence for and against the amyloid cascade hypothesis in AD and provides suggestions for future directions.

## 1. Introduction

Alzheimer's disease (AD), which is characterized by progressive deterioration in cognition, function, and behavior, places a considerable burden on western societies. It is the sixth leading cause of all deaths and the fifth leading cause of death in persons aged ≥65 years. To date, an estimated 5.4 million Americans have AD, but due to the baby boom generation, the incidence in 2050 is expected to reach a million persons per year, resulting in a total estimated prevalence of 11 to 16 million affected persons. 

**Figure 1:**
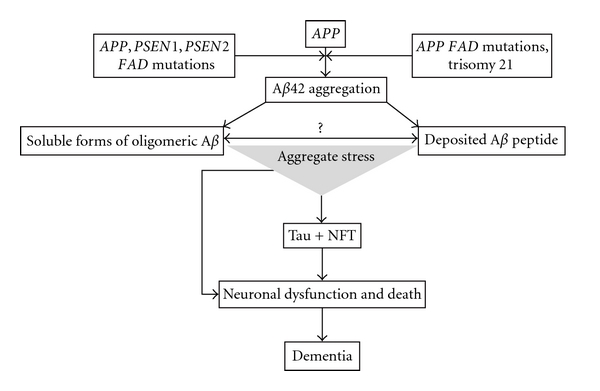
Amyloid cascade hypothesis.

Since the first description of presenile dementia by Alois Alzheimer in 1907 [1], senile plaques (SPs) and neurofibrillary tangles (NFTs) are considered the key pathological hallmarks of AD [2]. The identification of *β*-amyloid (A*β*) in SPs [3] and genetic studies that identified mutations in the amyloid precursor protein (*APP*) [4], presenilin 1 (*PSEN1*), and presenilin 2 (*PSEN2*) genes [5, 6] leading to the accumulation of A*β* and early-onset familial dementia [4, 5, 7], resulted in the formulation of the “Amyloid Cascade Hypothesis” (ACH; Figure 1) [8, 9]. According to the ACH, the deposition of A*β* is the initial pathological trigger in the disease, which subsequently leads to the formation of NFTs, neuronal cell death and dementia. While there is considerable evidence supporting this hypothesis, there are observations that seem to be inconsistent. This paper summarizes the current evidence for and against the amyloid cascade in AD.

## 2. Amyloid Cascade Hypothesis

As described above, two key observations resulted in the original formulation of the ACH (Figure 1). First, the detection of A*β* as a main constituent of the SPs [3] and second mutations of the *APP* [4], *PSEN1*, and *PSEN2* genes [5, 6], which were found in families with early-onset AD (FAD, disease onset < 60 years). As a consequence of these observations, the presence of A*β* within SPs was interpreted as an effect of these mutations that subsequently leads to cell death and dementia. Since FAD has—except the earlier onset—a similar phenotype to late-onset AD, it was assumed that this amyloid deposition could explain the pathogenesis of all types of AD.

## 3. Evidence from Studies on the Formation of A***β*** and Tau

There are two major objections regarding the ACH as originally formulated. First, SPs and NFTs may be reactive products resulting from neurodegeneration in AD rather than being its cause, and, second, it remains unclear whether and how the deposition of A*β* leads to the formation of NFTs. 

### 3.1. A*β* and Tau as Reactive Processes

 In persons who suffered from head trauma, APP is found with pathological features similar to AD in neuronal perikarya and in dystrophic neurites surrounding A*β* deposits [10]. In addition, there is evidence that neurons in the medial temporal lobe secrete APP and display increased APP immunoreactivity [11]. These findings suggest that increased expression of APP in head trauma cases may be an acute-phase response to neuronal injury [12], which in turn leads to increased A*β* deposition. This notion is supported by the observation that the different morphological forms of A*β* deposits, including diffuse, primitive, and classic deposits, contain acute phase proteins such as complement factors and *α*-antichymotrypsin [13]. Consequently, it has been proposed that, in AD, APP may be a reaction to the disease process in order to help maintain cell function, neuronal growth, and survival [14]. The putative neurotrophic action of APP is supported by the observation that it shares structural features with the precursor for epidermal growth factor [14]. Finally, there is also evidence that NFTs may form as a neuronal response to injury [15].

There are also findings from animal studies suggesting that the formation of A*β* and NFT may be reactive. In rats, both experimental damage or chemically induced lesions of the nucleus basalis can elevate cortical APP, and intrathecal or intraparenchymal injections of toxins can induce APP in hippocampal neurons, suggesting that the generation of APP could be a specific response to loss of functional innervation of the cortex [16, 17]. Denervation of the dopamine pathways and septal lesions affecting both the cholinergic system and *γ*-aminobutyric acid (GABA) neurons projecting to the dentate gyrus can result in a loss of dendritic microtubule-associated protein 2 (MAP2) and the appearance of tau-immunoreactive dentate gyrus granule cells [18]. Thus, denervation can cause transsynaptic changes in dentate gyrus neurons, and these alterations may represent an intermediate step to NFTs formation.

### 3.2. Relation of the Formation of NFT to A*β*

SPs and NFTs cluster in a significant proportion of cortical areas but they seem to be distributed independently of each other [19]. SP and NFTs also seem to occur temporally separated; in the entorhinal cortex the occurrence of NFTs may in fact precede the occurrence of SPs [20]. This spatial and temporal separation may suggest that they are pathogenically disconnected.

However, evidence for an effect of A*β* on the formation of NFT comes from transgenic experiments. The presence of *APP* mutations alone or in combination with *PSEN1* mutations seems to induce A*β* deposits in normal brain and some degree of hyperphosphorylated tau in neurites [21] although it does not appear to induce tau pathology or a significant inflammatory response. These findings are consistent with studies in which fetal rat hippocampal neurons and human cortical neurons treated with fibrielar A*β* display an increased degree of tau phosphorylation [22] providing additional evidence that amyloid fibril formation might alter the phosphorylation state of tau, which in turn results in the loss of microtubule-binding capacity. Other studies showed that A*β*_25–35_ can induce the aggregation of tau proteins and that a decrease in aggregation of A*β* was induced by tau peptides [23]. Thus, aggregation of tau may be associated with disassembly of A*β*, which could explain the lack of spatial correlation of the SPs and NFTs [19]. Finally, the notion of an impact of A*β* on NFT formation is supported by studies in *APP*-transgenic mice reporting that a reduction in endogenous levels of tau can ameliorate some of the behavioral and other deficits that are mediated by A*β* [24, 25] and by the discovery that mutations in the tau gene cause autosomal dominant frontotemporal lobe dementia with a tau pathology similar to the tau pathology seen in AD but without the appearance of A*β* plaques [26]. Both these observations seem to place tau pathology downstream of amyloid-*β* pathology.

## 4. Evidence from Genetic Studies

In particular the genes identified in the late-onset form of the disease provide support for the ACH. In general, these genes are not inherited in a Mendelian but a sporadic fashion. However, first-degree relatives of patients with late-onset AD have twice the expected life time risk of this disease compared to persons without an affected first-degree relative, and late-onset AD is more frequent among monozygotic than dizygotic cotwins, suggesting a substantial genetic contribution to this form of the disease.

The apolipoprotein E (*APOE*) gene, which was identified as the first susceptibility gene for late-onset AD, is the major genetic risk factor (population attributable risk: *∼*20%) [27, 28]. Each *APOE-*ε*4* allele lowers the age at onset in a dose-dependent fashion [27]. How the different APOE proteins mediate their effects in AD is not fully clarified, but there is compelling evidence by PDAPP transgenic mice models indicating that APOE mediates the clearance of amyloid-*β* [29], with the APOE2, APOE3, and APOE4 isoforms being increasingly less effective [30]. Consistent with this notion, the presence of an APOE-*ε*4 allele is associated with a higher A*β* burden in the brains of LOAD patients [31, 32], suggesting that APOE interacts with A*β* by enhancing its deposition in plaques. In various ethnic groups, two haplotypes in the sortilin-related receptor (*SORL1*) gene associated with LOAD were identified [33–37]. *SORL1* is involved in trafficking of APP from the cell surface to the golgi-endoplasmic reticulum complex and *γ*-secretase processing of APP [34, 38, 39], also in line with the ACH. Recent large-scale GWA studies performed primarily in samples and populations of European ancestry detected genetic variants associated with AD in complement component (3b/4b) receptor 1 (*CR1*), clusterin (*CLU, APOJ*), bridging integrator 1 (*BIN1*), phosphatidylinositol-binding clathrin assembly protein (*PICALM*), EPH receptor A1 (*EPHA1*), CD33 molecule (*CD33*), membrane-spanning 4-domains, subfamily A, members 4 and 6E (*MS4A4/MS4A6E*), CD2-associated protein (*CD2AP*), and ATP-binding cassette, subfamily A, member 7 (*ABCA7*) [40–42]. While these genes remain to undergo functional validation, they are functionally plausible and also largely consistent with the ACH. Similar and additive to *APOE*, *CLU* encodes an apolipoprotein and acts as an A*β* chaperone, regulating the conversion of A*β* to insoluble forms and A*β* toxicity thereby promoting amyloid plaque formation [43]. ABCA7 is involved in the efflux of lipids from cells to lipoprotein particles, such as APOE and CLU, and in addition regulates APP processing and inhibits *β*-amyloid secretion [44]. There is evidence that CR1 may contribute to A*β* clearance by complement activation [45]. CD2AP, CD33, BIN1, and PICALM are involved in endocytosis (CME), and a recent study [46] showed that several of these factors involved in endocytosis modify A*β* toxicity in glutamatergic neurons of* Caenorhabditis elegans* and in primary rat cortical neurons. In yeast, A*β* impaired the endocytic trafficking of a plasma membrane receptor, which was ameliorated by endocytic pathway factors identified in the yeast screen also providing substantial evidence for a link between A*β*, endocytosis, and human AD [46]. In summary, convincing evidence for an A*β*-related mechanism exists for all of these identified LOAD genes, providing a substantial amount of support for the ACH in AD.

## 5. Evidence from Clinical Trials Targeting A***β*** and Tau

The drugs currently used to treat AD (i.e., cholinesterase inhibitors, NMDA receptor antagonists, and antipsychotic drugs) have limited therapeutic value. New, potentially disease-modifying, therapeutic approaches are targeting A*β* and tau protein. Driven by the ACH, there are currently four main therapeutic approaches: (a) reducing the generation of A*β*, (b) facilitating the clearance of A*β*, (c) preventing the aggregation of A*β* and destabilizing A*β* oligomers, and (d) drugs targeting tau [47]. Drugs classes include active and passive immunization directed against A*β*, compounds that interfere with the secretases regulating A*β* generation from APP, drugs to prevent A*β* aggregation and destabilize A*β* oligomers, and drugs targeting tau protein.

### 5.1. Active and Passive Immunization

 Active and passive immunizations were developed to inhibit generation of toxic A*β* aggregates and to remove soluble and aggregated A*β*. At least three different immune-mediated mechanisms can promote A*β* removal: solubilization by antibody binding to A*β*, phagocytosis of A*β* by microglia, and A*β* extraction from the brain by plasma antibodies.

In phase II randomized controlled trials (RCTs) of active immunization of patients with mild-to-moderate AD with the anti-A*β* vaccine AN-1792 (QS-21) most but not all participants developed significant A*β*-antibody titres [48, 49] and there was evidence of memory and function improvement and reduced CSF tau concentrations in patients with increased IgG titres [48, 49]. However, in the first trial patients immunized with AN-1792 had a greater brain atrophy rate on MRI than did patients given placebo possibly because of amyloid removal and cerebral fluid shifts. In addition, several patients developed meningoencephalitis due to a T-cell response. In the follow-up trial, brain volume loss in antibody responders was not different from that in patients receiving placebo, and no further cases of meningoencephalitis were found [49]. Responders maintained low, but detectable, anti-AN-1792 antibody titres at about 4.6 years after immunization and had significantly reduced functional decline compared with placebo-treated patients [49]. In addition, immunization with anti-AN-1792 antibody could completely remove amyloid plaques as determined by postmortem assessment although patients still had end-stage dementia symptoms before death.

In order to avoid neuroinflammation and neurotoxicity, new vaccines that selectively target B-cell epitopes have been developed. CAD-106, which consists of the immunodrug carrier Qb coupled with a fragment of the A*β*_1–6_ peptide, could in animal studies induce A*β*-specific antibodies and reduce amyloid accumulation without stimulating T cells. In patients with mild-to-moderate AD, CAD-106 induced a substantial anti-A*β* IgG response and was well tolerated [50], confirmatory phase II RCTs are ongoing (NCT01097096, NCT01023685, NCT00795418, NCT00956410, and NCT00733863). ACC-001 is an A*β*_1–6_ fragment derived from the N-terminal B cell epitope of A*β* and conjugated to the mutated diphtheria toxin protein CRM19. It is being studied in phase II RCTs (NCT00479557, NCT01284387, NCT01227564, NCT00498602, NCT00752232, NCT00955409, NCT01238991, NCT00960531, NCT00959192). ACI-24 is a vaccine that contains A*β*_1–15_ closely apposed to the surface of the liposome. It reduced brain amyloid load and restored memory deficits in mice [51] and is entering a phase II RCT. Vaccines that are currently being tested in phase I RCTs are V-950 (NCT00464334; an aluminium-containing adjuvant with or without ISCOMATRIX (CSL Behring, PA, USA, a biological adjuvant of saponin, cholesterol, and phospholipids) and UB-311 (NCT00965588), a vaccine in which the immunogen A*β*_1–14_ is associated with the UBITh peptide (United Biomedical, NY, USA) and a mineral salt suspension adjuvant [52].

Affitopes, which are short peptides mimicking parts of native A*β*_1–42_, represent an alternative active immunization strategy. The affitopes AD-01 and AD-02 target the N-terminal A*β* fragment and both had disease-modifying properties in animal models of AD [53]. Results of recent phase I RCTs indicate that both are safe and well tolerated (NCT00495417, NCT00633841, and NCT00711139) [53]. Affitope AD-02 recently progressed to phase II clinical testing (NCT01117818).

Passive immunotherapy is based on monoclonal antibodies or polyclonal immunoglobulins targeting A*β* to promote its clearance. Animal studies have shown that anti-A*β* antibodies can prevent oligomer formation and reduce brain amyloid load with improvement in cognitive functions [54]. Several monoclonal antibodies are currently being tested: bapineuzumab (AAB-001), solanezumab (LY-2062430), PF-04360365, GSK-933776, R-1450 (RO-4909832), and MABT-5102A. A phase II RCT of bapineuzumab in patients with mild-to-moderate AD that had a follow-up period of longer than 18 months reported no significant effects on the primary measures of cognition or activities of daily living, as measured in prespecified within-dose cohort analyses. However, post hoc analyses of clinical and neuroimaging data from all dose cohorts showed nonsignificant improvements in cognitive endpoints and signs of efficacy in *APOE* **ɛ** *4* noncarriers [55]. Phase III studies are ongoing, including separate RCTs for *APOE* **ɛ** *4* carriers and non-carriers (NCT00574132, NCT00996918, NCT00998764, NCT00667810, NCT00575055, NCT00676143, and NCT00937352). Solanezumab, a monoclonal antibody that targets specifically soluble A*β*, promotes A*β* clearance from the brain through the blood. In a phase II RCT, there was a correlation between total plasma A*β*_1–42_ after treatment (dose-dependent increase), baseline amyloid plaque burden shown by single-photon emission CT scanning, and a dose-dependent increase in unbound CSF A*β*_1–42_, suggesting that solanezumab might mobilize A*β*_1–42_ from plaques and might normalize soluble CSF A*β*_1–42_ in patients with AD [56]. Consequently, two phase III RCTs have been initiated (NCT00905372, NCT00904683, NCT01127633). PF-04360365 is a modified IgG2 antibody that binds to the C terminus of A*β*_1–40_. Preliminary results on a single-dose regimen indicate that this antibody is well tolerated in patients with AD [57]. Currently, two phase II RCTs of multiple doses are ongoing (NCT00722046 and NCT00945672). GSK-933776, R-1450 (RO-4909832), and MABT-5102A are monoclonal antibodies that target A*β* and have been tested in patients with AD in phase I and phase II trials (NCT01424436, NCT00459550, NCT01224106, NCT00531804, NCT00736775, NCT00997919, NCT01343966, and NCT01397578).

Passive immunization [58] can also be achieved by intravenous infusion of immunoglobulins (IVIg), from healthy donors, which include naturally occurring polyclonal anti-A*β* antibodies. IVIg is already approved as therapy for immune deficiency, with good safety and tolerability evidence. In two small studies, short-term immunoglobulin administration in patients with AD was well tolerated, promoted a decrease of total A*β* CSF concentrations, and increased plasma total A*β* concentrations [59, 60], with evidence of improvement or stabilization of cognitive functions. Preliminary data from a phase II RCT confirmed the positive effects on cognition [61], a phase III study is ongoing (NCT00818662). In summary, the RCTs on active and passive immunization agents consistently show an effect on amyloid clearance, and several but not all phase II RCTs show promising effects on cognition.

### 5.2. Drugs to Reduce A*β* Generation from APP

BACE1 (*β*-secretase) initiates the amyloidogenic pathway. Pioglitazone and rosiglitazone are thiazolidinediones and drugs commonly used to treat type II diabetes. They happen to act as BACE1 inhibitors through stimulating the nuclear peroxisome proliferator-activated receptor **γ** (PPAR**γ**). Activation of PPAR**γ** receptors, in turn, can suppress expression of BACE1 and APP and can promote APP degradation by increasing its ubiquitination [62]. In addition to their effects on BACE1, therapeutic effects of PPAR*γ* agonists in AD could be caused by their effect on insulin action. Both rosiglitazone and pioglitazone increase peripheral insulin sensitivity and reduce concentrations of insulin. Insulin, in turn, competes with A*β* for degradation by the insulin-degrading enzyme [62].

There are only few phase III RCTs, which likely reflects the difficulty in development of BACE1 targeting agents. BACE1 has many substrates including several with physiologically important functions such as neuregulin-1 that is involved in myelination, and drugs must cross the blood-brain barrier in order to modulate BACE1 function. Pioglitazone can cross the blood-brain barrier although whether rosiglitazone can reach the CNS in human beings is unclear [62]. Out of the RCTs that have explored the effects of pioglitazone and rosiglitazone on cognition in patients with AD or MCI (NCT00982202, NCT00736996, NCT00550420, NCT00428090, NCT00348309, NCT00242593, NCT00265148, NCT00348140, NCT00334568, and NCT00490568), only three (NCT00982202, NCT00428090, and NCT00265148) have reported results to date, and these were negative [63]. Currently, several new *β*-secretase inhibitors are under investigation. Of these, CTS-21166, an orally administered compound, was well tolerated and reduced plasma A*β* concentrations in mice [64] and has proceeded to phase I clinical testing [65].

Development of drugs targeting *γ*-secretase, the enzyme responsible for the final step in A*β* generation, presents challenges similar to those for *β*-secretase inhibitors as *γ*-secretase is one of the main complexes involved in intramembranous cleavage of several proteins, including APP, Notch receptor, and various neuronal substrates [66]. As a consequence, adverse effects of *γ*-secretase inhibitors include hematological and gastrointestinal toxicity, skin reactions, and changes to hair color, mainly caused by inhibition of the Notch signaling pathway, which is involved in cell differentiation.

Phase III trials for the Notch-inhibiting drug semagacestat failed. Preliminary findings showed that semagacestat not only failed to slow disease progression, but also was associated with worsening of clinical measures of cognition and the ability to perform activities of daily living and a higher incidence of skin cancer in the treatment group than the placebo group. However, several Notch-sparing *γ*-secretase inhibitors (second-generation inhibitors) are currently under development: begacestat was tested in a phase I RCT (NCT00959881) and BMS-708163 in two phase II RCTs in patients with prodromal or mild-to-moderate AD (NCT00810147 and NCT00890890). Begacestat reduced A*β* concentrations in the plasma (with delayed rebound) [67] but did not substantially affect CSF A*β*_1–40_, whereas BMS-708163 promoted a dose-dependent decrease of A*β*_1–40_ in the CSF [68]. Results from animal studies testing PF-3084014 showed decreases in A*β* in the plasma, CSF, and brain, without a rebound effect on plasma A*β* [69]. In a subsequent small phase I study, PF-3084014 promoted a dose-dependent reduction in plasma A*β* concentrations although effects on CSF concentrations were small [70]. NIC5–15, a naturally occurring monosaccharide found in many foods, can act as a Notch-sparing *γ*-secretase inhibitor and insulin sensitizer (i.e., it increases the sensitivity of the tissue to insulin). It is currently being tested in patients with AD in a phase II study (NCT00470418).

*γ*-secretase *modulators* can selectively block APP proteolysis without Notch-based adverse effects. A subset of nonsteroidal anti-inflammatory drugs (NSAIDs), including ibuprofen, indomethacin, and sulindac sulfide, bind to APP and act as *γ*-secretase modulators, decreasing A*β*_1–40_ and A*β*_1–42_ production, with increased generation of A*β*_1–38_ fragments. Among these compounds, known as selective *β*-amyloid-lowering agents (SALAs), tarenflurbil was tested in phase III RCTs in patients with mild AD but did not show clinical effects [71] possibly due to low *γ*-secretase modulator potency, poor CNS penetration, or inhibition of microglia-mediated A*β* clearance by residual NSAID activity. Another *γ*-secretase modulator, CHF-5074, reduced A*β* brain load and improved behavioral deficits in animals [72] and has reached phase II clinical testing (NCT01303744 and NCT01421056).

Upregulation of **α*-secretase* activity, leading to non-amyloidogenic cleavage of APP, can decrease A*β* formation and increase production of a potentially neuroprotective soluble domain (sAPP*α*) [73]. Several drugs can stimulate *α*-secretase (agonists of muscarinic, glutamate, and serotonin receptors; statins; oestrogens; testosterone; protein kinase C activators) and have been tested in clinical trials, but no conclusive results are available yet [74]. These **α*-secretase* modulators include Exebryl-1, which modulates *β*- and *α*-secretase activity causing substantial reduction of A*β* formation and accumulation in the mouse brain with memory improvements (a phase I RCT was approved in 2008) [75], Etazolate (EHT-0202), a selective GABA_A_ receptor modulator that stimulates neuronal *α*-secretase and increases sAPP*α* production [76] and has been recently tested in a phase II RCT in patients with mild-to-moderate AD (NCT00880412) [77], and Bryostatin-1, a macrocyclic lactone that can stimulate *α*-secretase by activating protein kinase C and promoting sAPP*α* secretion [78] reducing brain A*β*_1–40_ and A*β*_1–42_ and improving behavioral outcomes in mouse models of AD [78] (phase II study in process (NCT00606164)).

### 5.3. Drugs to Prevent A*β* Aggregation and Destabilize A*β* Oligomers

Compounds that inhibit A*β* aggregation or destabilize A*β* oligomeric species can act twofold: (a) either they bind to A*β* monomers thereby preventing oligomerization and allowing A*β* elimination, or (b) they react with A*β* oligomers thereby neutralizing their toxicity and promoting their clearance. They are chemically heterogeneous and also here the challenge is to develop agents that can cross the blood brain barrier and have low toxicity.

The first generation of nonpeptidic antiaggregates failed to fulfill these criteria. Tramiprosate (3APS), which maintains A*β* in the nonfibrillar state by binding to soluble form, showed negative results in the Alphase study, a phase III RCT [79] although previous experimental and phase II trials had been promising [80]. Although there are several possible reasons for this failure, including variability among study sites, differences in the treatment and control groups because of the concomitant treatment with cognitive-enhancing drugs, and low CNS bioavailability of the drug, a European phase III RCT with tramiprosate was terminated as a consequence of the negative findings.

Clioquinol (PBT1) inhibits A*β* aggregation through interfering with interactions between A*β*, copper, and zinc. Studies in Tg2576 mice and human volunteers showed that CQ entry into the brain is limited although upon brain entry it binds to amyloid plaques [81]. PBT1 showed positive results in phase II RCTs but further phase II/III studies were halted due to manufacturing toxicity issues [82]. The second-generation inhibitor, PBT2, has a greater blood-brain barrier permeability than does clioquinol, and animal experiments showed that PBT2 prevents A*β* oligomerization, promotes A*β* oligomer clearance, reduces soluble and insoluble brain A*β*, decreases plaque burden, and has positive effects on cognition [82]. A 12-week, phase II RCT in patients with mild AD, was consistent with these findings, PBT2 reduced A*β*_1–42_ CSF concentrations and improved executive function [83]. Scyllo-inositol (ELND-005) is an orally administered stereoisomer of inositol that can cross the blood-brain barrier using inositol transporters. By binding to A*β*, it modulates its misfolding, inhibits its aggregation and stimulates dissociation of aggregates. It was successful in animal studies, reducing brain concentrations of soluble and insoluble A*β*_1–40_ and A*β*_1–42_, plaque burden, synaptic loss, and glial inflammatory reaction and significantly improving spatial memory function [84]. It is currently being tested in phase II RCTs (NCT00568776 and NCT00934050). However, because of serious adverse events among patients in the two high-dose groups (1000 mg and 2000 mg twice daily), these doses have been removed from the RCT, and the study continues restricted to patients who are assigned the lower dose (250 mg twice daily) and placebo. Epigallocatechin-3-gallate (EGCg), a polyphenol from green tea, induces *α*-secretase and prevents A*β* aggregation in animals by directly binding to the unfolded peptide [85]. In addition, it modulates signal transduction pathways, expression of genes regulating cell survival and apoptosis, and mitochondrial function [85]. It is currently being tested in a phase II/III RCT in patients with early AD.

### 5.4. Drugs to Target Tau Protein

Tau is a cytoplasmatic protein that binds to tubulin during its polymerisation, stabilising microtubules. In AD, tau is abnormally phosphorylated, resulting in the generation of aggregates (neurofibrillary tangles) toxic to neurons. The hypothesis that tau pathology causes AD has been the main competitor of the amyloid hypothesis [86]. However, only one tau-directed compound (valproate; valproic acid) has so far reached phase III RCT, with disappointing results because there were no effects on cognition and functional status [87]. 

There are two main therapeutic approaches to target the tau protein: modulation of tau phosphorylation with inhibitors of tau-phosphorylating kinases and compounds that inhibit tau aggregation and/or promoting aggregate disassembly. The first approach is based on the observation that tau hyperphosphorylation and neurofibrillary tangle formation can be promoted by imbalanced activity of protein kinases (glycogen-synthase-kinase-3 (GSK3) and p70-S6-kinase) and the phosphatase PP2A [88]. GSK3 deregulation might have a role in AD pathogenesis because GSK3 is involved in tau and amyloid processing, cellular signaling, and gene transcription [88].

Both lithium and valproate, well known for the treatment of psychiatric disorders, inhibit GSK3, to reduce tau phosphorylation and prevent or reverse aspects of tauopathy in animal models [89]. Both drugs can also be neuroprotective by upregulating the antiapoptotic factor BCL2, inducing neurotrophic factors, and hindering A*β* toxicity [89]. However, a small RCT with lithium (10 weeks, including a 6-week titration phase) in patients with mild AD did not show any cognitive benefit or any change in CSF biomarkers, including phosphorylated tau, total tau, and A*β*_1–42_ [90]. 

The AD Cooperative Study (ADCS) of valproate was designed to determine whether chronic valproate treatment could delay the onset of behavioral symptoms in outpatients with mild-to-moderate AD; a secondary aim was to test whether valproate can delay cognitive and functional decline. No effects on cognition and functional status were reported, but incidence of agitation and psychosis seemed to be reduced [89]. 

Several GSK3 inhibitors are under development. NP-031112 (NP-12) is a thiadiazolidinone-derived compound, a non-ATP competitive inhibitor of GSK3, which can reduce brain concentrations of phosphorylated tau and amyloid deposition and prevent neuronal death and cognitive deficits in animals [91]. This drug has been tested in patients with AD in a phase II RCT (NCT00948259); no results have yet been published.

Methylthioninium chloride (methylene blue), a widely used histology dye, acts as a tau antiaggregate [92]. This compound also has antioxidant properties, enhances mitochondrial function [93], and was effective, alone and in combination with rivastigmine, in reversing learning deficits and hyoscine-induced memory impairments in animals [94]. Different doses of methylthioninium chloride (up to 100 mg) were tested in a phase II study in patients with moderate AD. The group given the 60 mg dose had improved cognitive function and, after 1 year, evidence of slower disease progression compared with placebo [95]. The ineffectiveness in the group on the 100 mg dose was attributed to drug formulation defects, limiting release. A new formulation (leuco-methylthioninium), with a higher bioavailability, was recently announced [96], and phase III RCTs are needed to confirm its safety and clinical efficacy.

Davunetide (AL-108, NAP), an intranasally administered, eight-aminoacid peptide fragment derived from the activity-dependent neuroprotective protein, and AL-208, an intravenous formulation of Davunetide, are being developed. Davunetide has been tested in animal models of AD and tauopathy, and its neuroprotective activity includes regulation of microtubule dynamics, as well as inhibition of tau hyperphosphorylation and protection against A*β* toxicity [97, 98]. Davunetide was studied in patients with amnestic mild cognitive impairment in a 12-week, phase II RCT and was safe and well tolerated and had positive effects on cognition [99], although confirmatory studies are needed.

Nicotinamide is the biologically active form of niacin (vitamin B3) and the precursor of coenzyme NAD+. Orally administered nicotinamide can prevent cognitive deficits in a mouse model of AD and can reduce brain concentrations of a species of phosphorylated tau (Thr231) that inhibits microtubule polymerization [100]. Furthermore, nicotinamide inhibits brain sirtuin deacetylase and upregulates acetyl-*α*-tubulin, protein p25, and MAP2c; all these interactions are associated with increased microtubule stabilization [100]. Nicotinamide has been used in several clinical studies, including RCTs in patients with neurodegenerative disorders, and is generally safe and well tolerated; a phase II RCT is ongoing in patients with mild-to-moderate AD (NCT00580931).

What do these trials tell us? Sadly, they leave little certainty. Amyloid immunization teaches us that we can massively reduce amyloid burden, but when administered late in the disease, it is not a miracle cure. It may have clinically relevant benefits and it may lead to better outcomes if it is given early in the disease or presymptomatically but we simply do not have data to address these issues.

## 6. Conclusions

Overall, there is substantial evidence supporting a role of the ACH in AD. However, the available results from RCTs are not in line with previous optimistic predictions of an imminent breakthrough in development of a disease-modifying therapy. To explain the disappointing results of several RCTs, researchers have highlighted various potential issues, both in drug choice and development programs.  Table 1 summarizes these and provides possible solutions. Clinical trials need to be organized in those in the very earliest stages of the disease. Whether this can be carried out genetically (e.g., by using E4 homozygotes) or by PIB imaging or some combination of both is not clear. Of course, it could be argued that even persons who show PIB signals are already too far down the disease progression for disease-modifying therapy and that treatment needs to be initiated even before this stage. Certainly, even those with mild AD have profound cell loss. In addition, it would be helpful to perform antiamyloid trials in individuals with *APP* and *PSEN* mutations or those with Down's syndrome as they provide the best test of the ACH hypothesis. Biomarker studies should be included in trial designs so that the researchers can form, as clearly as possible, informed opinions as to whether the drug has hit the proposed target.

However, in addition to implementing new guidelines in preclinical and clinical phases of drug development, several additional issues are key to validate the ACH and successfully develop therapeutic targets. From a molecular point of view, we need a focused effort to fully understand the functions of APP and A*β* and to answer the two key questions: does A*β* in fact influence tau phosphorylation and, if yes, does tau phosphorylation in fact lead to dementia? Second, we need to understand the nature of disease propagation: is permissive templating of A*β* [101, 102] and tau [103] the reason for both the characteristic neuroanatomy of the disease [104] and the reason that the disease seems to become self-propagating once it has started [105, 106]?. Finally, it makes sense to pursue other targets beyond A*β* as there is substantial evidence for additional potential pathways increasing disease susceptibility, among these lipid metabolism and inflammatory processes [107]. 

## Figures and Tables

**Table 1:** Issues of RCTs of AD.

Issue	Possible solution
*Subjects*	
Target group selection: patients with AD have various types of neuropathology (i.e., amyloid plaques, NFTs, infarcts, and Lewy bodies)	Criteria for identifying subgroups with more homogeneous biomarker evidence of AD pathology are needed to facilitate RCTs
In patients with mild-to-moderate AD, the disease could be too advanced for a disease-modifying effect of a specific drug (e.g., immunotherapy)	RCTs that include patients with early AD might enable detection of disease-modifying effects; investigation into which stage of the AD process a therapeutic strategy is more effective is warranted

*Agents*	
Choosing the right drug: compounds with positive results in preclinical and early clinical testing failed in large phase III RCTs, with costly losses (e.g., tramiprosate)	Robust proof-of-concept studies should be mandatory Investigators should take into account class efficacy
	Use of drug-related biomarkers in preclinical and early clinical stages can help to confirm the target engagement and to assure early withdrawal of ineffective drugs
Some RCTs were likely hindered by the inability to reach a therapeutic dosage (e.g., tarenflurbil) or short treatment duration	Optimization of drug dosage and treatment duration based on pharmacokinetics
Genetics: polymorphisms (e.g., APOE,) might affect drug response	Personalized therapeutic approach: considering genetic polymorphisms that affect drug response can help to optimize drug dosage (e.g., increased doses for individuals with a rapid metabolism)

*Outcome measurements*	
Measuring effects: many RCTs are developed according to the design of AChEI RCTs, an approach that has indicated the AChEI symptomatic effect but is not sensitive in detecting the efficacy of disease-modifying drugs, rating scales used may have low sensitivity for changes and/or the drug type assessed and these tools have a subjective component	Development and use of relevant, reliable, multidimensional measures for clinical (cognitive and functional) endpoints are key factors, as well the use of biomarkers (neuroimaging, CSF, or blood molecules) that reliably and quantitatively correlate with disease progression; collection of baseline data (clinical, biomarkers) that can be used as reference to interpret later findings is advisable; for early AD (i.e., mild cognitive impairment), self-rated and observer-rated assessments of activities of daily living, instrumental activities of daily living, and quality of life are recommended
Unreliable evaluation of patients by RCT raters	Adequate training and monitoring of RCT raters to maximize homogeneous recruitment of patients, reduce variance, and guarantee a more accurate rating; effective implementation of quality control on data at research sites

*Optimization of resources*	
Consistency: multicenter RCTs done in several countries can have cultural and linguistic issues with assessment scales (e.g., translation, validation), as well as infrastructure problems (technological disparities between centers)	Multicenter trials should use centers of excellence that are already experienced in RCTs to minimize intersite and intercountry variability
Unsuccessful preclinical and clinical studies are often not published leading to repetition of unsuccessful trials or errors	More collaboration between pharmaceutical companies and clinical researchers, with information sharing, can lead to more standardized RCT protocols, reduction of errors, and decreased costs
